# Progresses and challenges in *Strongyloides* spp. proteomics

**DOI:** 10.1098/rstb.2022.0447

**Published:** 2024-01-15

**Authors:** Natalia Tiberti, Marcello Manfredi, Chiara Piubelli, Dora Buonfrate

**Affiliations:** ^1^ Department of Infectious, Tropical Diseases and Microbiology, IRCCS Sacro Cuore Don Calabria Hospital, 37024 Negrar di Valpolicella (Verona), Italy; ^2^ Department of Translational Medicine, University of Piemonte Orientale, 28100 Novara, Italy

**Keywords:** strongyloidiasis, proteomics, excretory/secretory product, somatic proteome, immunoproteomics

## Abstract

The availability of high-quality data of helminth genomes provided over the past two decades has supported and accelerated large-scale ‘omics studies and, consequently, the achievement of a more in-depth molecular characterization of a number of pathogens. This has also involved *Strongyloides* spp. and since their genome was made available transcriptomics has been rather frequently applied to investigate gene expression regulation across their life cycle. *Strongyloides* proteomics characterization has instead been somehow neglected, with only a few reports performing high-throughput or targeted analyses associated with protein identification by tandem mass spectrometry. Such investigations are however necessary in order to discern important aspects associated with human strongyloidiasis, including understanding parasite biology and the mechanisms of host–parasite interaction, but also to identify novel diagnostic and therapeutic targets. In this review article, we will give an overview of the published proteomics studies investigating strongyloidiasis at different levels, spanning from the characterization of the somatic proteome and excretory/secretory products of different parasite stages to the investigation of potentially immunogenic proteins. Moreover, in the effort to try to start filling the current gap in host-proteomics, we will also present the first serum proteomics analysis in patients suffering from human strongyloidiasis.

This article is part of the Theo Murphy meeting issue ‘*Strongyloides*: omics to worm-free populations’.

## 1. Human strongyloidiasis and other *Strongyloides* spp.

Human strongyloidiasis by *Strongyloides stercoralis* is widely distributed across the world, with foci of current intense transmission in areas of poor sanitation and inadequate wastewater disposal [1]. Less prevalent species causing human infection are known to be endemic in Papua New Guinea (*Strongyloides fuelleborni kellyi)* and in parts of Africa (*Strongyloides fuelleborni fuelleborni*) [2].

Non-human primates, dogs and wild canids (and probably cats) can also be infected by *S. stercoralis* [3], thus the zoonotic potential is under investigation. Other animals may have distinct *Strongyloides* species; among the latter *S. ratti* and *S. venezuelensis,* parasites of rodents, have been widely used in laboratory experimental infections [4] both for research purposes and to retrieve crude extracts as antigens for the implementation of serological assays for the diagnosis of human strongyloidiasis [5].

Acute infection is seldom observed in tourists returning from endemic areas [6]. Clinical manifestations correlate with the larval migration across the body: immediately after skin penetration, infected individuals may present cutaneous signs and symptoms such as rash, pruritus and oedema at the portal of entry of the filariform infective larvae. Loeffler syndrome, characterized by cough and dyspnoea, follows a few days later, sometimes along with urticarial rash; eosinophilia is typically observed. Two–three weeks from infection, following the settlement of adult female worms in the gut, acute gastrointestinal symptoms (e.g. diarrhoea, abdominal pain) may occur [6,7].

Chronic infection, due to the autoinfective cycle, can lead to a wide range of clinical manifestations, from unapparent disease to severe systemic syndromes. Similarly to acute infection, symptoms usually involve skin, respiratory tract and intestine [2]. Eosinophilia is frequently observed, although the eosinophil count can be within the normal range or fluctuating [2]. Host–parasite interaction is usually regulated in immunocompetent individuals, while immunosuppression leads to an uncontrolled, accelerated autoinfection cycle, resulting in a dramatic increase in parasite load. This can cause mechanical obstruction with consequent worsening of clinical conditions (e.g. paralytic ileus, respiratory impairment); moreover, sepsis and/or meningitis by Gram negative bacteria have been associated with hyperinfection, contributing to increase the fatality rate. Further, larvae and even other parasite stages (adults and eggs) can disseminate all over the body. Risk factors that have been frequently associated with the development of hyperinfection/dissemination are human T-cell lymphotropic virus type1 (HTLV-1) infection, malignancies, treatment with steroids and other immunosuppressant drugs (such as biologics) [8].

*Strongyloides stercoralis* has a complex life cycle characterized by the alternation between free-living and parasitic life stages, in addition to its peculiar ability to undergo autoinfection within the human host [9]. The ability of this parasite to generate chronic long-lasting infections usually without apparent clinical manifestations [10] clearly indicates the establishment and efficient regulation of the interaction between the host and the parasite that should be discerned in order to achieve a more comprehensive knowledge of the pathophysiological mechanisms associated with human strongyloidiasis. *Strongyloides*, similar to most other helminths, is known to trigger a Th2 immune response, which should protect against hyperinfection. This response is associated with the production of type 2 cytokines and reduced Th1 and Th17 responses [2]. However, this classical type 2 profile was not observed in a cohort of elderly Italians with chronic strongyloidiasis lasting for decades and in the absence of re-exposure [11]. Mechanisms of disease tolerance might in fact establish during chronic strongyloidiasis as a defence tool for the host to limit damage associated with a persistently activated immune response. At the same time, the parasite could exploit this tolerance to ensure its survival within the host [12–14]. Although the specific mechanisms are still largely unknown, it is thus clear that the host–parasite interplay is crucial in the maintenance of parasitism during strongyloidiasis.

## 2. Insights into the somatic proteome of *Strongyloides* spp.

Compared to other helminth parasites, proteomics investigations of *Strongyloides* spp. have been largely under-reported. Recent technological advances in the field of proteomics offer the possibility of employing different strategies to achieve an adequate protein identification even from complex samples, including from multi-cellular organisms like helminths*.* These strategies encompass (i) the classical in-gel separation methods coupled with band/spot excision, in-gel digestion and subsequent protein identification; (ii) the analysis of protein suspensions by tandem mass spectrometry (LC-MS/MS) possibly, but not necessarily, coupled with different fractionation (e.g. off-gel electrophoresis, strong cation exchange) and/or labelling methods. The more recent advent of data-independent acquisition (DIA) mass spectrometry [15] is currently allowing the quantitative analysis of low-abundant samples without the need for in-gel protein separation, even though such an approach has yet to be applied to the study of *Strongyloides* spp.

The first report describing the proteomics analysis of *S. stercoralis* infective larvae (iL3) somatic proteome was published in 2010, when Marcilla and colleagues employed a controlled proteolytic cleavage to identify proteins associated with the parasite surface as well as from whole larval lysate [16]. Although the number of identifications (i.e. 26 proteins) was limited by the lack of a reference genome, the different digestion treatments performed in the study allowed the identification of proteins potentially associated with the larval surface, since they were detected after short digestion of whole larvae with trypsin. Importantly, some of the identified proteins—such as galectins, 14-3-3 and heat shock proteins—were subsequently found to be associated with *S. venezuelensis* iL3 somatic proteome and with *S. ratti* excretory/secretory products (ESPs) (discussed in the next paragraph) [17,18]. A more comprehensive coverage of the somatic proteome was indeed later obtained for *S. ratti* parasitic and free-living adult females [19] and for *S. venezuelensis* iL3 [18], taking advantage of the availability of a reference genome for *S. ratti* and of a draft genome for *S. venezuelensis* [19]. The tandem mass spectrometry approach applied to the study of *S. ratti* provided a catalogue encompassing 1266 proteins from adult females. A comparative analysis revealed extensive differences in the protein expression between free living (Ff) and parasitic female (Pf) worms, since 569 proteins were reported as upregulated in Pf and 409 in Ff. Even though the number of differential proteins was particularly elevated (maybe due to the applied statistics) and similar differences were not observed at the transcriptome level [19], the study clearly showed the complexity of an in depth proteomics characterization of helminth parasites. Indeed, in order to comprehensively characterize these parasites, the different life stages and the different sub-proteomes should be investigated and ideally compared.

A large number of somatic proteins (i.e. 877) was also identified from the shot-gun proteomics analysis of *S. venezuelensis* iL3. The most abundant components of *S. venezuelensis* iL3 somatic proteome included enzymes (particularly oxidoreductases), structural proteins, CAP domain proteins, heat shock proteins, proteases and protease inhibitors [18]. It is noteworthy that some of these proteins were proposed to be potentially involved in *Strongyloides* parasitism in comparative genomics and transcriptomics analyses [19,20]. Very similar results were recently obtained by our group through the analysis of *S. stercoralis* iL3 proteome [21]. Indeed, we identified 430 proteins from *S. stercoralis* iL3 isolated from a clinical sample. Through the use of a semi-automated strategy, we annotated our dataset for gene ontology terms and protein domains, including uncharacterized proteins that represented 43% of our identifications. In agreement with previous reports, we confirmed the high representation within iL3 somatic proteome of proteins potentially associated with *Strongyloides* parasitism, including oxidoreductases, peptidases and peptidase inhibitors, proteins containing SCP/TAPS/CAP or thioredoxin domains and proteins belonging to cysteine-rich secretory, transthyretin-like or peptidase protein families, galectins and transthyretin-like proteins [21]. Importantly, we also identified with good confidence Ss-NIE, a protein employed in several in-house and commercial kits for the serodiagnosis of human strongyloidiasis [22–25].

Taken together, these proteomic catalogues could contribute to building a core proteome shared between *Strongyloides* species and/or life stages. Such analyses should thus be expanded in order to cover different life stages (especially the parasitic ones) and the different species (table 1).

**Table 1.** Summary of published studies dealing with proteomics investigations of *Strongyloides* spp. Pf, parasitic female; Ff, free-living female; Fl, free-living larva; HSPs, heat shock proteins.

*Strongyloides* species	parasite source	life stage	research question	experimental method	no. of identified proteins	no. of unique peptides for protein id. (≥)	ref.
**somatic proteome**							
*S. stercoralis*	human stools	iL3	identification of iL3 somatic proteome	controlled proteolytic cleavage + LC-MS/MS	26	2	[16]
*S. stercoralis*	human stools	iL3	identification of iL3 somatic proteome	shot-gun LC-MS/MS	430	2	[21]
*S. ratti*	stools and intestine from infected rats	Pf	comparative analysis of somatic proteome from parasitic and free living females	shot-gun LC-MS/MS	Pf: 857	1	[19]
		Ff			Ff: 697		
*S. venezuelensis*	stools from infected rats	iL3	identification of iL3 somatic proteome	shot-gun LC-MS/MS	877^a^	1	[18]
**excretory/secretory products (ESPs)**							
*S. ratti*	stools and intestine from infected rats	Pf	comparative analysis of ESPs from different life stages	in-gel separation and protein identification by LC-MS/MS	iL3-ESP: 450	2	[17]
		iL3			Pf-ESP: 335		
		Fl			Fl-ESP: 217		
*S. ratti*	intestine from infected rats	Pf	identification of small heat shock proteins (HSPs) in ESPs	in-gel separation and protein identification by LC-MS/MS	Na^c^	2	[26]
*S. venezuelensis*	stools from infected rats	iL3	identification of immuno-reactive bands from iL3 ESPs with patients' serum	in-gel separation and protein identification by LC-MS/MS of immuno-reactive bands	74^b^	1	[27]
*S. venezuelensis*	Am	iL3	comparative analysis of ESPs from different life stages	in-gel separation and protein identification by LC-MS/MS	iL3-ESP: 436	unknown	[28]
		Pf			Pf-ESP: 196		
**immunogenic proteins^d^**							
*S. stercoralis*	human stools	iL3	identification of immunogenic iL3 proteins	immunoblot and protein identification by LC-MS/MS	2	2	[29]
*S. stercoralis*	human stools	iL3	identification of immunogenic iL3 proteins	2-DE + immunoblot and protein identification by LC-MS/MS	20	2	[30]
*S. stercoralis*	n/a	n/a	*in silico* prediction of potentially immunogenic proteins	pipeline of algorithms applied to *S. stercoralis* UniProt proteome	34 candidates	n/a	[31]
*S. stercoralis*	human stools	iL3	*in silico* prediction of potentially immunogenic proteins	B-cell epitope prediction applied to an experimental proteomic dataset	9 candidates	n/a	[21]
*S. venezuelensis*	stools from infected rats	iL3	identification of immunogenic iL3 proteins	immunoblot and protein identification by LC-MS/MS	11	2	[32]

^a^The number of proteins identified with at least two unique peptides is 766.

^b^Overall number of proteins identified using different experimental conditions.

^c^Protein identification analysis only focussed on HSP proteins.

^d^For this category, the number of identified proteins corresponds to those identified from immuno-reactive bands/spots or predicted *in silico*.

## 3. Proteomics analysis of *Strongyloides* spp. excretory/secretory products

Helminth parasites have developed different mechanisms in order to survive and to adapt to the adverse conditions within their hosts. It is now evident that the release of ESPs plays a central role in this host–parasite interaction [33]. ESPs include both waste molecules excreted in the environment and biomolecules actively secreted through specific pathways by live helminths [34]. Since they are active at the host–parasite interface and in the parasite-mediated modulation of the host immune system [33,35], ESPs represent a unique source of potential targets for the development of diagnostic tools and vaccines, as well as for therapeutic interventions for other pathological conditions, including auto-immune disorders [36–38]. For these reasons, they are raising significant interest within the scientific community and the release of ESPs has already been proven and investigated in multiple helminth species, including hookworms [39], *Anisakis* spp. [40], whipworms [41], filarial nematodes [42] and *Strongyloides* spp.

ESPs include a variety of biomolecules, i.e. small molecules, lipids and carbohydrates [39]; however, a predominant functional role is played by proteins. The prediction of secreted proteins using bioinformatic tools is often incomplete, as it is now clear that mechanisms of unconventional protein secretion exist and only a limited portion of secreted proteins possess the signal peptides driving the classical endoplasmic reticulum–Golgi secretion pathway [43]. The experimental investigation of ESP protein composition using high-throughput approaches is thus essential to gain novel insights into the immunomodulatory properties of parasite secretome.

As mentioned above, ESPs are also being investigated in *Strongyloides* spp. to reveal novel aspects of parasite biology as well as their immunomodulatory properties. Nonetheless, apart from a few studies dating back to the ‘80s and ‘90s [44,45], ESPs from *S. stercoralis* are yet to be investigated in depth using up-to-date high throughput approaches. More pieces of evidence have instead been collected using the close relatives *S. venezuelensis* and *S. ratti* (table 1).

A comparative proteomics analysis revealed a stage-dependent protein composition of *S. ratti* ESPs since different in-gel protein profiles were observed, further confirmed by protein identification by tandem mass spectrometry. In particular, L3 infective larvae (iL3) showed the highest number of stage-specific proteins (*n* = 196) compared to Pf (*n* = 79) and free-living larvae (Fl, *n* = 35) [17]. It is worth mentioning that those iL3 had not been exposed to a host, thus they were presumably developmentally arrested. Given that protein release within ESPs was proven to occur via an excretion/secretion process and not as a consequence of cell death or damage [17], the presence of proteins specific to iL3 ESPs—including proteases—might support their role in the establishment of the infection or in the progression throughout the parasite life cycle. Nonetheless, it would be particularly interesting to explore and compare ESP proteins released by iL3 external to the host or at the very early stage of host invasion. Such analysis could in fact contribute in revealing additional factors specifically associated with iL3 mechanisms of infection and parasitism. Beside stage-specific proteins, 23.8% of the identified proteins were common to the three life stages analysed, including heat-shock proteins, galectins, enzymes, fatty acid binding protein and structural proteins [17]. These shared proteins could contribute to building a core ESP proteome from *Strongyloides* spp*.*, even though the variability in larval maintenance conditions across studies should be considered as a possible bias of these analyses.

The untargeted proteomics characterization was also performed on ESPs released by *S. venezuelensis* iL3 and Pf [28]. Some iL3 ESP proteins had already been proposed to be potentially associated with parasitism in *S. stercoralis* genomic and transcriptomics analyses [19], including astacins, astacin-like peptidases and SCP/TAPS proteins. Indeed, although their molecular function is still largely unknown, these proteins have been proposed to be involved in parasite migration through the host as well as in immunomodulation [46,47].

Younis *et al.* used tandem mass spectrometry to investigate specific protein categories, namely small heat shock proteins (sHSPs), within ESPs of *S. ratti* parasitic female worms. Through an extensive bioinformatics analysis, they assigned two sequences as putative HSP-17 (then named Sra-HSP-17.1 and Sra-HSP-17.2), which were further characterized to establish their biological role in the host–pathogen interaction [26]. Indeed, Sra-HSP-17s were found to be highly immunogenic in immunized rats as well as being recognized by the antibodies present in *S. stercoralis*-infected patients [26].

An important component of helminth-derived ESPs is represented by extracellular vesicles (EVs or exosome-like vesicles). Evidence supporting the active release of EVs from different helminth species both *in vitro* and *in vivo* has now been collected [48], however EV release in *Strongyloides* has so far been neglected. These vesicular elements carrying biological material are key players in inter-parasite and host–pathogen interactions [48–51]. Described for the first time in 2012 in the trematodes *Echinostoma caproni* and *Fasciola hepatica* [52], they have subsequently raised large interest within the helminth scientific community and helminth-derived EVs have now been described in at least 17 helminth species [53]. In parallel, the characterization of their protein cargo has become of importance in order to reveal novel potential biomarkers for disease diagnosis and control [53,54].

Despite their potential, the study of helminth-derived EVs presents numerous challenges and a standardization of the working procedures was recently deemed necessary, prompting the publication of recommended guidelines for their study [55]. Although experimental data documenting *Strongyloides*-derived EVs are not yet available, Gonzales *et al*. employed bioinformatics tools to predict the presence of numerous proteins secreted within EVs among those identified in *S. venezuelensis* iL3 ESPs [27], concluding that EVs release is also likely to occur in *Strongyloides*.

## 4. Immunoproteomics

The ultimate goal of most proteomics studies applied to *Strongyloides* spp., performed either on the somatic proteome or on the secretome, is the identification of protein targets for the development of novel serological assays. Indeed, although serological assays represent the most accurate diagnostic methods among those available, their performance still needs to be improved, both as screening and as diagnostic tools [56]. Compared to classical parasitological methods, they present lower specificity also due to possible cross-reactions with other helminths [5]. The sensitivity of serological methods can instead be very high (up to 83–95%), even though it can decrease in cases of immunosuppression or at the very early stage of infection [5]. Another important issue associated with strongyloidiasis serodiagnosis is the possibility of false positive results due to a delay in seroreversion after treatment. The identification of markers whose detection is specifically indicative of an active infection would be particularly useful in developing novel diagnostic tests allowing this issue to be overcome. It is thus clear that current diagnostic methods need to be improved to achieve more reliable disease management and control.

Two main paths have been undertaken by the scientific community to try to improve current serological tests using immunoproteomics approaches: (1) the identification of antigenic fractions from *S. stercoralis, S. venezuelensis* or *S. ratti* for the development of serological assays based on homologous or heterologous crude extracts; (2) the identification of specific *S. stercoralis* immunogenic proteins to be used in recombinant antigen-based tests (table 1). Both approaches have their own advantages and disadvantages. Indeed, although they are easily obtainable, since crude extracts rely on the use of host laboratory animals for their supply, they are associated with inter-batch variability and usually present a higher level of cross-reaction with other helminthiases compared with recombinant proteins. On the other hand, the employment of ELISA assays based on recombinant proteins reduces the inter-assay variability and the issues associated with antigen supply; nonetheless, the identification and production of *S. stercoralis* recombinant immunogenic proteins is costly and the development of a high-performance immunoassay is challenging and time-consuming.

The utility of *S. ratti* somatic larval crude extract is well recognized, since it is employed in a commercial serological ELISA kit used for the diagnosis of human strongyloidiasis (*Strongyloides ratti* IgG ELISA, Bordier Affinity Products, CH). *S. ratti* ESPs have also been reported as immunoreactive with serum from infected rats as well as with serum from patients with human strongyloidiasis, and this immunoreactivity was shown to be life stage-dependent, since Pf- and iL3-ESPs displayed stronger reactivity compared to Fl-ESPs [17]. Even though only a limited number of samples was investigated and only total IgG antibodies were measured, Soblik and colleagues provided the first evidence of ESP immunoreactivity with patients' serum, further supporting the importance of this antigenic fraction at the host–parasite interface.

As previously mentioned, *S. venezuelensis* has also been evaluated in a number of studies as a heterologous source of immunogenic antigens. The immunoreactivity of *S. venezuelensis* iL3 somatic protein extract (both soluble and membrane fractions) was evaluated using immunoblotting coupled with protein identification of immunoreactive bands. A 30–40 kDa immunoreactive antigenic fraction was shown to contain metabolic enzymes, cytoskeletal proteins and galectins [32]. However, also based on previous results from *S. ratti*, the potential of ESPs as a source of immunoreactive antigens soon became clear and in 2017 Cunha and colleagues published the first report evaluating the utility of employing *S. venezuelensis* ESPs [57]. Importantly, through a direct comparison they showed for the first time the superior immunoreactivity of *S. venezuelensis* ESPs compared to the somatic extract when tested with serum from infected patients and uninfected controls. ESPs showed lower cross-reactivity with serum of patients suffering from other helminthiases compared to the somatic extract and was able to detect antibodies in both immunocompetent and immunocompromised *Strongyloides*-positive individuals [57]. Those results were further confirmed by Roldán Gonzáles *et al.* [58]*,* who compared the diagnostic accuracy of three different antigenic preparations from *S. venezuelensis*, namely soluble somatic fraction, membrane somatic fraction and ESPs. Once again, when tested for immunoreactivity with human serum, ESPs showed the highest accuracy for discriminating between *Strongyloides*-positive sera and negative controls, while displaying the lowest rate of cross-reactivity with serum from patients suffering from other helminthiases. Even though those comparative studies proved the higher potential of the ESP fraction for serodiagnosis, they also highlighted the need to identify by mass spectrometry the specific immunogenic antigens in relation to the development of novel diagnostic tools. Even if superior in accuracy compared to somatic extracts, the use of ‘crude’ ESPs in a diagnostic tool is still limited by the variability of the antigenic source, the need for animal models as a source of parasites and the lack of standardized procedures for ESP collection. Indeed, ESPs are obtained through the maintenance of larvae *in vitro,* and different maintenance conditions—in terms of incubation time, medium and larval density—have been reported in the literature. This technical variability seems to affect the larval metabolic state and, as a consequence, the ESP pattern [58] since differences in ESP proteomics composition were observed in larvae maintained in PBS or RPMI [27]. The identification of immunoreactive proteins thus seems essential to overcome this variability, as was done in iL3-ESP from *S. venezuelensis.* Mass spectrometry in fact identified arginine kinase as a protein band that is highly immunoreactive with patients' serum (93% sensitivity and 100% specificity) and that has no cross-reactivity with serum samples from patients with other helminthiases [27].

An ideal serological test should however be based on homologous antigens in order to maximize both sensitivity and specificity. The first studies investigating the immune-recognition of proteins from *S. stercoralis* somatic extract by patients’ serum date back to the ‘90s, when the detection of different polypeptide bands derived from *S. stercoralis* infective larvae by patients' antibodies was shown, even though band detection was not followed by protein identification [59–61]. The first protein identification of *S. stercoralis* immuno-reactive bands using LC-MS/MS was performed by Rodpai and colleagues, who identified a 26 kDa band (corresponding to 14-3-3) and a 29 kDa band (corresponding to ADP/ATP translocase 4) from iL3 as highly immunogenic since they were recognized by patients' serum with SE/SP of 90%/76.5% and 80%/92.2%, respectively [29]. However, only a limited number of clinical samples were tested (i.e. *n* = 10) and both bands presented cross-reaction, although limited, with other parasitic infections. The same research group later employed two-dimensional gel electrophoresis (2-DE) combined with immunoblot and protein identification by mass spectrometry to partly corroborate those findings. Indeed, using a more extensive separation technique they confirmed the immunogenic nature of 14-3-3 and detected additional protein spots as immunoreactive with patients' serum, among which galectin and enolase are worth mentioning [30]. Both these proteins have already been identified in multiple studies as candidate diagnostic markers both in *Strongyloides* spp. as well as in other parasitic helminths [17,27,28]. Moreover, enolase has already been proposed as a vaccine candidate against *Ascaris suum* [62], while galectins are involved in the initiation of the host immune response [35,63–65].

*In silico* approaches nowadays represent important alternatives for the prediction of potentially immunogenic proteins, and immuno-informatics can be efficiently employed to screen larger protein datasets that are now available, either as predicted by the draft genomes or as experimentally obtained. Recently, such an approach has also been applied to *S. stercoralis*. In particular, a reverse approach using different algorithms and web-based tools was employed by Culma [31] to predict potentially immunogenic peptides from the UniProt *S. stercoralis* proteome (12.851 entries). The adopted pipeline, encompassing prediction of subcellular localization, helices, allergenicity, B- and T-cell epitopes, and evaluation of homology and physicochemical properties, highlighted 34 proteins as the most promising candidates for the future development of vaccines or diagnostics. Interestingly, none of these proteins was found by our group in a recent study using different B-cell epitope prediction tools to highlight potentially immunogenic proteins from *S. stercoralis* iL3 somatic proteome [21]. This apparent discrepancy could be explained by the fact that in our study we focussed the prediction analysis only on proteins experimentally identified from iL3 larvae, while Culma used the entire proteome of *S. stercoralis* available in UniProt, encompassing proteins from all developmental stages [31]. Importantly, among the candidates proposed in our study we highlighted galectin, confirming previous results [30] and Ss-NIE. This latter protein is currently employed in the most promising serological assay based on recombinant proteins [22–25].

## 5. Proteomics to study the host response to the infection

The evaluation of the host response through the high-throughput analysis of a host's body fluids or tissues during strongyloidiasis has yet to be performed. Such analysis could however be particularly useful in revealing novel facets of the pathological mechanisms associated with human strongyloidiasis, especially when applied in comparative studies. Indeed, this strategy has already been successfully applied to the study of other helminthiases, including schistosomiasis, fasciolosis and echinococcosis, to identify proteins or biological processes altered in the pathological state compared to uninfected controls, during the disease progression or in response to treatment [66–76]. The most commonly investigated samples are plasma/serum and plasma/serum-derived EVs (either from animal models or from patients), even though also tissue proteomics has been described [69,70]. Importantly, a number of studies identified parasite proteins within the host's systemic or vesicular proteome, supporting the relevance of host proteomics also in revealing novel diagnostic markers [67,68,71,72,74,75].

Considering strongyloidiasis, many studies have investigated the systemic host response to the infection through the measurement of specific circulating factors, especially cytokines [11,77–83]. However, an untargeted high-throughput approach has yet to be undertaken.

In our group, we recently employed SWATH-MS proteomics to identify and quantify serum proteins from *n* = 5 patients suffering from long-lasting chronic strongyloidiasis (electronic supplementary material). The investigated population represented a sub-group of a cohort of elderly Italian subjects suffering from autochthonous strongyloidiasis that was previously studied to evaluate the systemic levels of selected cytokines, chemokines and immune factors [11]. Here, we sought to expand those investigations through the untargeted analysis of the systemic proteome using LC-MS/MS. Serum samples taken on admission (baseline – before treatment) and 6 and 12 months post-treatment with ivermectin were analysed, together with samples from age- and gender-matched uninfected controls (electronic supplementary material, tables S1 and S2). All patients whose serum was analysed signed a written informed consent for the donation of their biological samples for research purposes. The study received ethical approval by the Ethical Committee of Verona and Rovigo provinces under the protocol no. 23730 (24 April 2019).

Our analysis quantified 208 serum proteins, 17 of which were found to be significantly differentially abundant between infected subjects at baseline compared to uninfected controls (i.e. *p*-value < 0.05; fold change, FC ≤ 0.83 or FC ≥ 1.2). Considering post-treatment samples, 9 and 8 proteins were significantly differentially abundant at 6 and 12 months after treatment compared to baseline, respectively (figure 1*a,b*; electronic supplementary material, table S3). Among differential proteins, coagulation factor 5 (FA5) was significantly increased in infected patients compared to controls, and its profile reverted with a significant decrease in abundance both 6 and 12 months post-treatment (figure 1*c*). Cyclin-dependent kinase 10 (CDK10) and Ubinuclein-1 (UBN1) showed increased serum abundance after treatment (both at 6 and 12 months) compared to baseline, while epidermal growth factor (EGF)-containing fibulin-like extracellular matrix protein 1 (FBLN3) decreased after treatment (figure 1*c*). It is noteable that 3 proteins associated with blood coagulation and platelet activation (namely FA5, von Willberand factor and fibrinogen) were significantly increased in infected patients compared to controls. Despite this observation, the overall number of modulated proteins was not large enough to allow performance of a formal pathway analysis to highlight potentially modulated biological processes associated with chronic long-lasting strongyloidiasis. This could suggest a fine-tuning modulation in protein expression occurring at the systemic level in patients infected for such a long time, in agreement with the establishment of mechanisms of disease tolerance in these patients [12], allowing the infection to persist without developing a significant pathology. In order to highlight these small changes in protein abundances, a larger number of samples should probably be analysed and a comparison with samples from patients from endemic areas should be performed. Such patients should in fact bear infections of more recent acquisition and should probably be more frequently re-exposed to the pathogen. We did not identify any parasite proteins in our samples, but this is not surprising since at this chronic stage of infection parasites are confined to the intestinal tract, with females reproducing by parthenogenesis within the intestinal mucosa [9].

**Figure 1.**
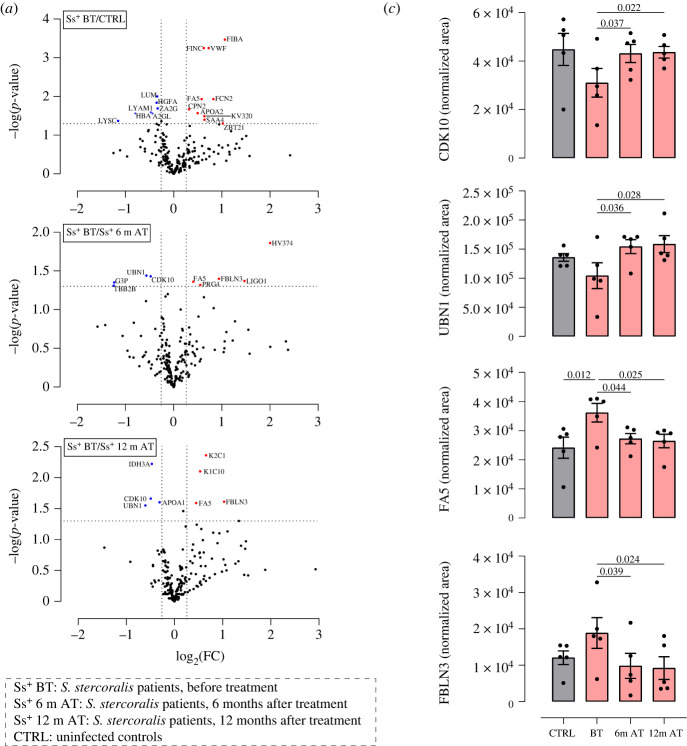
Results of host serum proteomics analysis applied to investigate samples from patients with strongyloidiasis (taken before treatment (BT) and 6 and 12 months after treatment) and uninfected controls. For each group *n* = 5 samples were analysed. (*a*) Volcano plot showing the differential abundance of the quantified proteins in the different samples. The dotted line on the *y*-axes represents 0.05 *p*-value threshold, while the dotted lines on the *x*-axes represent 0.83 and 1.2 FC thresholds. (*b*) (*overleaf*) Table summarizing proteins significantly differentially abundant in at least one of the three analysed comparisons. For each protein, the *p*-value and the fold change are reported. Orange values: *p*-value < 0.05; blue values: FC ≤ 0.83 or ≥1.2. (*c*) Detailed results for four proteins showing differential abundance in at least two of the assessed comparisons. The bar charts show the mean normalized area values for each group; error bars represent the standard error. Grey, uninfected controls; pink: patients with strongyloidiasis. All plots were generated using GraphPad Prism 8 (GraphPad Software LLC, MA, USA).

**Figure 1.**
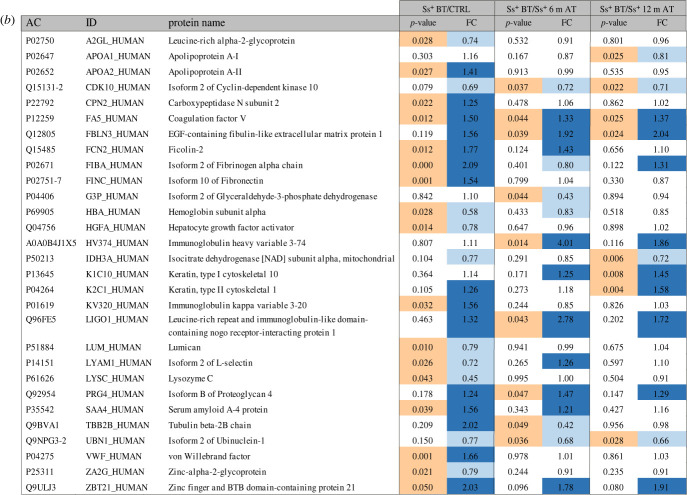
(*Continued*.)

If extended to a larger number of samples (including other patient groups) and/or to other matrices, such as serum-derived EVs, host proteomics analyses might reveal novel aspects associated with the pathophysiology of chronic strongyloidiasis that will be fundamental to understanding the mechanisms of maintenance of parasitism and tolerance.

## 6. Conclusion and future perspectives

Proteomics studies of *Strongyloides* spp. have for long time been limited by the lack of reference or draft genomes, which have only been available since 2016 [19]. The availability of high-quality data of helminth genomes is supporting and accelerating large-scale gene expression studies using ‘omics approaches, including proteomics. Indeed, proteomics approaches can be particularly useful in studying different aspects of helminthiases of clinical importance, including: (i) to gain novel insights into parasite biology; (ii) to discern the mechanisms of host–parasite interaction; (iii) to identify novel diagnostic and therapeutic targets as well as potential vaccine candidates. An important limitation associated with *S. stercoralis* proteomics is however the lack of a manually annotated reference proteome. Indeed, the reference proteome currently available in UniProtKB/TrEMBL contains 12900 entries (organism ID 6248, as of 18th of July 2023) corresponding to unreviewed protein sequences automatically annotated. In order to make comparative proteomics data more trustworthy and impactful, it is still necessary to improve current annotation at both the genome and proteome levels. The technological progress in mass spectrometry observed in the past decades nowadays allows us to generate large amounts of experimental data, which however cannot be fully evaluated in the biological context due to the lack of a manually annotated proteome.

Working with *Strongyloides* spp. presents other significant challenges mainly related to their complex life cycles. The investigation of clinical isolates, obtained either from patients’ samples or from wild animals, should probably be favoured over laboratory strains maintained in laboratory animals. It has in fact been hypothesized that laboratory strains may not be entirely representative of the parasite's behaviour, as the different environments are likely to impact on their biological features and developmental strategies [84]. In the post-genomics era, parasitology research has significantly grown, bringing our knowledge of parasite biology and the mechanisms of host–parasite interaction to a new level. Pioneering work has been done on *Strongyloides* spp., using CRISPR/Cas9-mediated mutagenesis, for instance [85], and this nematode still remains the most studied model for genetic transformation [86,87], highlighting the potential of this parasite as a model nematode to study the mechanisms of parasitism. Nonetheless, the potential differences between clinical/wild isolates and laboratory strains should be kept in mind and both comparative and functional studies should thus be focussed as much as possible on clinical/wild isolates.

The complex life cycle of *Strongyloides* spp. still hampers the *in vitro* growth of this parasite. Although ambitious, the development of an *in vitro* model able to reproduce the parasitic life cycle would represent a major breakthrough in *Strongyloides* research, as it would provide the proper amount of parasitic material to perform in depth ‘omics and functional studies while reducing the use of laboratory animals.

Large proteomics datasets are now being made available for different *Strongyloides* spp., for different life stages and for different fractions. Even though there are still some gaps to be filled, it is now necessary to bring proteomics studies a further step forward through functional analysis of specific targets of interest. Indeed, we should take advantage of *Strongyloides* as a nematode model for genetic transformation to explore the mechanisms of parasitism, of host–pathogen interaction and of host response at the molecular level in order to obtain novel tools to fight strongyloidiasis.

## Data Availability

The mass spectrometry proteomics data can be accessed from the ProteomeXchange Consortium via the PRIDE partner repository with the dataset identifier PXD041218: https://proteomecentral.proteomexchange.org/cgi/GetDataset?ID=PXD041218 [88]. The data are provided in electronic supplementary material [89].

## References

[1] Buonfrate D et al. 2020 The global prevalence of *Strongyloides stercoralis* infection. Pathogens **9**, 468. (10.3390/pathogens9060468) 32545787 PMC7349647

[2] Nutman TB. 2017 Human infection with *Strongyloides stercoralis* and other related *Strongyloides* species. Parasitology **144**, 263-273. (10.1017/S0031182016000834) 27181117 PMC5563389

[3] Thamsborg SM, Ketzis J, Horii Y, Matthews JB. 2017 *Strongyloides* spp. infections of veterinary importance. Parasitology **144**, 274-284. (10.1017/S0031182016001116) 27374886

[4] Viney M, Kikuchi T. 2017 *Strongyloides* *ratti* and *S. venezuelensis* - rodent models of *Strongyloides* infection. Parasitology **144**, 285-294. (10.1017/S0031182016000020) 26935155 PMC5364835

[5] Buonfrate D, Tamarozzi F, Paradies P, Watts MR, Bradbury RS, Bisoffi Z. 2022 The diagnosis of human and companion animal *Strongyloides stercoralis* infection: challenges and solutions. A scoping review. Adv. Parasitol. **118**, 1-84. (10.1016/bs.apar.2022.07.001) 36088083

[6] Angheben A et al. 2011 Acute strongyloidiasis in Italian tourists returning from Southeast Asia. J. Travel Med. **18**, 138-140. (10.1111/j.1708-8305.2010.00496.x) 21366799

[7] Alabi A, Boggild AK, Bitnun A. 2017 Acute strongyloidiasis in a child recently returned from vacation in Cuba. CMAJ **189**, E1416-E1420. (10.1503/cmaj.170464) 29158455 PMC5698030

[8] Buonfrate D, Requena-Mendez A, Angheben A, Munoz J, Gobbi F, Van Den Ende J, Bisoffi Z. 2013 Severe strongyloidiasis: a systematic review of case reports. BMC Infect. Dis. **8**, 78. (10.1186/1471-2334-13-78) PMC3598958 23394259

[9] Viney ME, Lok JB. 2015 The biology of *Strongyloides* spp. WormBook 1-17. (10.1895/wormbook.1.141.2) PMC5402216 26183912

[10] Abrescia FF et al. 2009 Reemergence of strongyloidiasis, northern Italy. Emerg. Infect. Dis. **15**, 1531-1533. (10.3201/eid1509.090191) 19788836 PMC2819861

[11] Tiberti N, Buonfrate D, Carbone C, Piro G, Bisoffi Z, Piubelli C. 2020 Systemic profile of immune factors in an elderly Italian population affected by chronic strongyloidiasis. Parasites Vectors **13**, 515. (10.1186/s13071-020-04391-w) 33059754 PMC7559927

[12] Allen JE, Maizels RM. 2011 Diversity and dialogue in immunity to helminths. Nat. Rev. Immunol. **11**, 375-388. (10.1038/nri2992) 21610741

[13] King IL, Li Y. 2018 Host–parasite interactions promote disease tolerance to intestinal helminth infection. Front. Immunol. **9**, 2128. (10.3389/fimmu.2018.02128) 30298071 PMC6160735

[14] Mishra PK, Palma M, Bleich D, Loke P, Gause WC. 2014 Systemic impact of intestinal helminth infections. Mucosal Immunol. **7**, 753-762. (10.1038/mi.2014.23) 24736234

[15] Gillet LC, Navarro P, Tate S, Rost H, Selevsek N, Reiter L, Bonner R, Aebersold R. 2012 Targeted data extraction of the MS/MS spectra generated by data-independent acquisition: a new concept for consistent and accurate proteome analysis. Mol. Cell. Proteomics: MCP **11**, O111 016717. (10.1074/mcp.O111.016717) PMC3433915 22261725

[16] Marcilla A et al. 2010 Proteomic analysis of *Strongyloides stercoralis* L3 larvae. Parasitology **137**, 1577-1583. (10.1017/S0031182010000314) 20388238

[17] Soblik H, Younis AE, Mitreva M, Renard BY, Kirchner M, Geisinger F, Steen H, Brattig NW. 2011 Life cycle stage-resolved proteomic analysis of the excretome/secretome from *Strongyloides ratti*--identification of stage-specific proteases. Mol. Cell. Proteomics: MCP **10**, M111 010157. (10.1074/mcp.M111.010157) PMC3237078 21964353

[18] Fonseca PDM et al. 2020 Shotgun proteomics of *Strongyloides venezuelensis* infective third stage larvae: insights into host–parasite interaction and novel targets for diagnostics. Mol. Biochem. Parasitol. **235**, 111249. (10.1016/j.molbiopara.2019.111249) 31881239

[19] Hunt VL et al. 2016 The genomic basis of parasitism in the *Strongyloides* clade of nematodes. Nat. Genet. **48**, 299-307. (10.1038/ng.3495) 26829753 PMC4948059

[20] Hunt VL, Tsai IJ, Selkirk ME, Viney M. 2017 The genome of *Strongyloides* spp. gives insights into protein families with a putative role in nematode parasitism. Parasitology **144**, 343-358. (10.1017/S0031182016001554) 27618747

[21] Dishnica K, Piubelli C, Manfredi M, Kondaveeti RT, Longoni SS, Degani M, Buonfrate D, Giorgetti A, Tiberti N. 2023 Novel insights into the somatic proteome of *Strongyloides stercoralis* infective third-stage larvae. Parasites Vectors **16**, 45. (10.1186/s13071-023-05675-7) 36721249 PMC9890704

[22] Sears WJ, Nutman TB. 2022 Strongy detect: preliminary validation of a prototype recombinant Ss-NIE/Ss-IR Based ELISA to detect *Strongyloides stercoralis* infection. PLoS Neglected Trop. Dis. **16**, e0010126. (10.1371/journal.pntd.0010126) PMC8789141 35077470

[23] Yunus MH, Arifin N, Balachandra D, Anuar NS, Noordin R. 2019 Lateral flow dipstick test for Serodiagnosis of Strongyloidiasis. Am. J. Trop. Med. Hyg. **101**, 432-435. *Strongyloides stercoralis* protein and/or corresponding DNA and RNA sequences for application in diagnosis' filed in Malaysia (PI 2015002836) and the United States, No. 15/778430. (10.4269/ajtmh.19-0053) 31218996 PMC6685572

[24] Balachandra D, Rahumatullah A, Lim TS, Mustafa FH, Ahmad H, Anuar NS, Noordin R. 2021 A new antigen detection ELISA for the diagnosis of *Strongyloides* infection. Acta Trop. **221**, 105986. (10.1016/j.actatropica.2021.105986) 34058161

[25] Rascoe LN, Price C, Shin SH, McAuliffe I, Priest JW, Handali S. 2015 Development of Ss-NIE-1 recombinant antigen based assays for immunodiagnosis of strongyloidiasis. PLoS Neglected Trop. Dis. **9**, e0003694. (10.1371/journal.pntd.0003694) PMC4393093 25860665

[26] Younis AE et al. 2011 Stage-specific excretory-secretory small heat shock proteins from the parasitic nematode *Strongyloides* *ratti*--putative links to host's intestinal mucosal defense system. FEBS J. **278**, 3319-3336. (10.1111/j.1742-4658.2011.08248.x) 21762402 PMC3718022

[27] Roldan Gonzales WH, Coelho GR, Pimenta DC, de Paula FM, Gryschek RCB. 2022 Proteomic analysis of the excretory-secretory products from *Strongyloides* *venezuelensis* infective larvae: new insights for the immunodiagnosis of human strongyloidiasis. Parasitol. Res. **121**, 3155-3170. (10.1007/s00436-022-07636-y) 36044090

[28] Maeda Y, Palomares-Rius JE, Hino A, Afrin T, Mondal SI, Nakatake A, Maruyama H, Kikuchi T. 2019 Secretome analysis of *Strongyloides* *venezuelensis* parasitic stages reveals that soluble and insoluble proteins are involved in its parasitism. Parasites Vectors **12**, 21. (10.1186/s13071-018-3266-x) 30626426 PMC6327390

[29] Rodpai R, Intapan PM, Thanchomnang T, Sanpool O, Janwan P, Laummaunwai P, Wongkham C, Insawang T, Maleewong W. 2016 *Strongyloides stercoralis* diagnostic polypeptides for human strongyloidiasis and their proteomic analysis. Parasitol. Res. **115**, 4007-4012. (10.1007/s00436-016-5170-7) 27312043

[30] Rodpai R, Intapan PM, Thanchomnang T, Sanpool O, Janwan P, Laummaunwai P, Wongkham C, Insawang T, Maleewong W. 2017 Identification of antigenic proteins in *Strongyloides stercoralis* by proteomic analysis. Parasitol. Res. **116**, 1687-1693. (10.1007/s00436-017-5443-9) 28455628

[31] Culma MF. 2021 *Strongyloides stercoralis* proteome: a reverse approach to the identification of potential immunogenic candidates. Microb. Pathog. **152**, 104545. (10.1016/j.micpath.2020.104545) 33091578

[32] Corral MA et al. 2017 Potential immunological markers for diagnosis of human strongyloidiasis using heterologous antigens. Parasitology **144**, 124-130. (10.1017/S0031182016001645) 27894367

[33] Maizels RM, Smits HH, McSorley HJ. 2018 Modulation of host immunity by helminths: the expanding repertoire of parasite effector molecules. Immunity **49**, 801-818. (10.1016/j.immuni.2018.10.016) 30462997 PMC6269126

[34] Harnett W. 2014 Secretory products of helminth parasites as immunomodulators. Mol. Biochem. Parasitol. **195**, 130-136. (10.1016/j.molbiopara.2014.03.007) 24704440

[35] Hewitson JP, Grainger JR, Maizels RM. 2009 Helminth immunoregulation: the role of parasite secreted proteins in modulating host immunity. Mol. Biochem. Parasitol. **167**, 1-11. (10.1016/j.molbiopara.2009.04.008) 19406170 PMC2706953

[36] Joshi P, Mishra PKK. 2022 Functional diversity of the excretory/secretory proteins of nematode parasites. Acta Parasitol. **67**, 619-627. (10.1007/s11686-022-00523-7) 35113339

[37] Helmby H. 2015 Human helminth therapy to treat inflammatory disorders - where do we stand? BMC Immunol. **16**, 12. (10.1186/s12865-015-0074-3) 25884706 PMC4374592

[38] Loukas A, Maizels RM, Hotez PJ. 2021 The yin and yang of human soil-transmitted helminth infections. Int. J. Parasitol. **51**, 1243-1253. (10.1016/j.ijpara.2021.11.001) 34774540 PMC9145206

[39] Abuzeid AMI, Zhou X, Huang Y, Li G. 2020 Twenty-five-year research progress in hookworm excretory/secretory products. Parasites Vectors **13**, 136. (10.1186/s13071-020-04010-8) 32171305 PMC7071665

[40] Mehrdana F, Buchmann K. 2017 Excretory/secretory products of anisakid nematodes: biological and pathological roles. Acta Vet. Scand. **59**, 42. (10.1186/s13028-017-0310-3) 28645306 PMC5482935

[41] Shears RK, Grencis RK. 2022 Whipworm secretions and their roles in host-parasite interactions. Parasites Vectors **15**, 348. (10.1186/s13071-022-05483-5) 36175934 PMC9524059

[42] Hotterbeekx A, Perneel J, Vieri MK, Colebunders R, Kumar-Singh S. 2021 The Secretome of filarial nematodes and its role in host-parasite interactions and pathogenicity in onchocerciasis-associated epilepsy. Front. Cell. Infect. Microbiol. **11**, 662766. (10.3389/fcimb.2021.662766) 33996633 PMC8113626

[43] Filaquier A, Marin P, Parmentier ML, Villeneuve J. 2022 Roads and hubs of unconventional protein secretion. Curr. Opin. Cell Biol. **75**, 102072. (10.1016/j.ceb.2022.02.006) 35305454

[44] Brindley PJ, Gam AA, Pearce EJ, Poindexter RW, Neva FA. 1988 Antigens from the surface and excretions/secretions of the filariform larva of *Strongyloides* *stercoralis*. Mol. Biochem. Parasitol. **28**, 171-180. (10.1016/0166-6851(88)90001-1) 3386679

[45] McKerrow JH, Brindley P, Brown M, Gam AA, Staunton C, Neva FA. 1990 *Strongyloides* *stercoralis*: identification of a protease that facilitates penetration of skin by the infective larvae. Exp. Parasitol. **70**, 134-143. (10.1016/0014-4894(90)90094-S) 2137091

[46] Varatharajalu R, Parandaman V, Ndao M, Andersen JF, Neva FA. 2011 *Strongyloides stercoralis* excretory/secretory protein strongylastacin specifically recognized by IgE antibodies in infected human sera. Microbiol. Immunol. **55**, 115-122. (10.1111/j.1348-0421.2010.00289.x) 21204942 PMC6117823

[47] Cantacessi C, Gasser RB. 2012 SCP/TAPS proteins in helminths—where to from now? Mol. Cell. Probes **26**, 54-59. (10.1016/j.mcp.2011.10.001) 22005034

[48] Sanchez-Lopez CM, Trelis M, Bernal D, Marcilla A. 2021 Overview of the interaction of helminth extracellular vesicles with the host and their potential functions and biological applications. Mol. Immunol. **134**, 228-235. (10.1016/j.molimm.2021.03.020) 33836351

[49] Eichenberger RM, Sotillo J, Loukas A. 2018 Immunobiology of parasitic worm extracellular vesicles. Immunol. Cell Biol. **96**, 704-713. (10.1111/imcb.12171) 29808496

[50] Tritten L, Geary TG. 2018 Helminth extracellular vesicles in host–parasite interactions. Curr. Opin. Microbiol. **46**, 73-79. (10.1016/j.mib.2018.08.002) 30172862

[51] Drurey C, Maizels RM. 2021 Helminth extracellular vesicles: interactions with the host immune system. Mol. Immunol. **137**, 124-133. (10.1016/j.molimm.2021.06.017) 34246032 PMC8636279

[52] Marcilla A et al. 2012 Extracellular vesicles from parasitic helminths contain specific excretory/secretory proteins and are internalized in intestinal host cells. PLoS ONE **7**, e45974. (10.1371/journal.pone.0045974) 23029346 PMC3454434

[53] Sotillo J, Robinson MW, Kimber MJ, Cucher M, Ancarola ME, Nejsum P, Marcilla A, Eichenberger RM, Tritten L. 2020 The protein and microRNA cargo of extracellular vesicles from parasitic helminths – current status and research priorities. Int. J. Parasitol. **50**, 635-645. (10.1016/j.ijpara.2020.04.010) 32652128

[54] Montano KJ, Loukas A, Sotillo J. 2021 Proteomic approaches to drive advances in helminth extracellular vesicle research. Mol. Immunol. **131**, 1-5. (10.1016/j.molimm.2020.12.030) 33440289

[55] White R et al. 2023 Special considerations for studies of extracellular vesicles from parasitic helminths: a community-led roadmap to increase rigour and reproducibility. J. Extracellular Vesicles **12**, e12298. (10.1002/jev2.12298) 36604533 PMC9816087

[56] Bisoffi Z et al. 2014 Diagnostic accuracy of five serologic tests for *Strongyloides stercoralis* infection. PLoS Neglected Trop. Dis. **8**, e2640. (10.1371/journal.pntd.0002640) PMC3890421 24427320

[57] Cunha RA, de Carvalho EFG, de Sousa JEN, Costa-Cruz JM. 2017 Excretory/secretory antigens of *Strongyloides* *venezuelensis* applied to IgG detection in human strongyloidosis. Parasitol. Int. **66**, 671-676. (10.1016/j.parint.2017.07.001) 28705595

[58] Roldan Gonzales WH, Meisel D, de Paula FM, Gryschek RCB. 2021 Diagnostic accuracy of somatic and excretory-secretory antigens from *Strongyloides* *venezuelensis* infective larvae for the immunodiagnosis of human strongyloidiasis. Parasitology **148**, 1522-1527. (10.1017/S0031182021001207) 35060455 PMC11010145

[59] Sato Y, Inoue F, Matsuyama R, Shiroma Y. 1990 Immunoblot analysis of antibodies in human strongyloidiasis. Trans. R. Soc. Trop. Med. Hyg. **84**, 403-406. (10.1016/0035-9203(90)90337-E) 2260175

[60] Conway DJ, Bailey JW, Lindo JF, Robinson RD, Bundy DA, Bianco AE. 1993 Serum IgG reactivity with 41-, 31-, and 28-kDa larval proteins of *Strongyloides stercoralis* in individuals with strongyloidiasis. J. Infect. Dis. **168**, 784-787. (10.1093/infdis/168.3.784) 8354924

[61] Sudre AP, Siqueira RC, Barreto MG, Peralta RH, Macedo HW, Peralta JM. 2007 Identification of a 26-kDa protein fraction as an important antigen for application in the immunodiagnosis of strongyloidiasis. Parasitol. Res. **101**, 1117-1123. (10.1007/s00436-007-0596-6) 17569087

[62] Chen N et al. 2012 *Ascaris suum* enolase is a potential vaccine candidate against ascariasis. Vaccine **30**, 3478-3482. (10.1016/j.vaccine.2012.02.075) 22465737

[63] Donskow-Lysoniewska K, Maruszewska-Cheruiyot M, Stear M. 2021 The interaction of host and nematode galectins influences the outcome of gastrointestinal nematode infections. Parasitology **148**, 648-654. (10.1017/S003118202100007X) 33461629 PMC11010190

[64] Jaleta TG, Lok JB. 2019 Advances in the molecular and cellular biology of *Strongyloides* spp. Curr. Trop. Med/. Rep. **6**, 161-178. (10.1007/s40475-019-00186-x) 31929961 PMC6953981

[65] Loghry HJ, Sondjaja NA, Minkler SJ, Kimber MJ. 2022 Secreted filarial nematode galectins modulate host immune cells. Front. Immunol. **13**, 952104. (10.3389/fimmu.2022.952104) 36032131 PMC9402972

[66] Goncalves-Silva G, Vieira L, Cosenza-Contreras M, Souza AFP, Costa DC, Castro-Borges W. 2022 Profiling the serum proteome during *Schistosoma mansoni* infection in the BALB/c mice: a focus on the altered lipid metabolism as a key modulator of host-parasite interactions. Front. Immunol. **13**, 955049. (10.3389/fimmu.2022.955049) 36119112 PMC9471378

[67] Guo X et al. 2022 Proteomic profiling of serum extracellular vesicles identifies diagnostic markers for echinococcosis. PLoS Neglected Trop. Dis. **16**, e0010814. (10.1371/journal.pntd.0010814) PMC9581430 36206314

[68] Bi NN, Zhao S, Zhang JF, Cheng Y, Zuo CY, Yang GL, Yang K. 2021 Proteomics investigations of potential protein biomarkers in sera of rabbits infected with *Schistosoma japonicum*. Front. Cell. Infect. Microbiol. **11**, 784279. (10.3389/fcimb.2021.784279) 35004354 PMC8729878

[69] Osakunor DNM, Ishida K, Lamanna OK, Rossi M, Dwomoh L, Hsieh MH. 2022 Host tissue proteomics reveal insights into the molecular basis of *Schistosoma haematobium*-induced bladder pathology. PLoS Neglected Trop. Dis. **16**, e0010176. (10.1371/journal.pntd.0010176) PMC8846513 35167594

[70] Becerro-Recio D, Serrat J, Lopez-Garcia M, Sotillo J, Simon F, Gonzalez-Miguel J, Siles-Lucas M. 2022 Proteomics coupled with *in vitro* model to study the early crosstalk occurring between newly excysted juveniles of *Fasciola hepatica* and host intestinal cells. PLoS Neglected Trop. Dis. **16**, e0010811. (10.1371/journal.pntd.0010811) PMC9555655 36223411

[71] Shi C et al. 2022 Proteomic analysis of plasma-derived extracellular vesicles from mice with *Echinococcus granulosus* at different infection stages and their immunomodulatory functions. Front. Cell. Infect. Microbiol. **12**, 805010. (10.3389/fcimb.2022.805010) 35360110 PMC8960237

[72] Kardoush MI, Ward BJ, Ndao M. 2016 Identification of candidate serum biomarkers for *Schistosoma mansoni* infected mice using multiple proteomic platforms. PLoS ONE **11**, e0154465. (10.1371/journal.pone.0154465) 27138990 PMC4854390

[73] Bexkens ML, van Gestel RA, van Breukelen B, Urbanus RT, Brouwers JF, Nieuwland R, Tielens AGM, Van Hellemond JJ. 2020 *Schistosoma mansoni* infection affects the proteome and lipidome of circulating extracellular vesicles in the host. Mol. Biochem. Parasitol. **238**, 111296. (10.1016/j.molbiopara.2020.111296) 32603736

[74] Fratini F et al. 2020 Proteomic analysis of plasma exosomes from Cystic Echinococcosis patients provides *in vivo* support for distinct immune response profiles in active vs inactive infection and suggests potential biomarkers. PLoS Neglected Trop. Dis. **14**, e0008586. (10.1371/journal.pntd.0008586) PMC7535053 33017416

[75] Wang W, Zhou X, Cui F, Shi C, Wang Y, Men Y, Zhao W, Zhao J. 2019 Proteomic analysis on exosomes derived from patients' sera infected with *Echinococcus granulosus*. Korean J. Parasitol. **57**, 489-497. (10.3347/kjp.2019.57.5.489) 31715689 PMC6851256

[76] Da'dara AA, Siddons G, Icaza M, Wang Q, Skelly PJ. 2017 How schistosomes alter the human serum proteome. Mol. Biochem. Parasitol. **215**, 40-46. (10.1016/j.molbiopara.2016.12.007) 28011341 PMC5474353

[77] Anuradha R, Munisankar S, Bhootra Y, Jagannathan J, Dolla C, Kumaran P, Shen K, Nutman TB, Babu S. 2016 Systemic Cytokine profiles in *Strongyloides stercoralis* infection and alterations following treatment. Infect. Immun. **84**, 425-431. (10.1128/IAI.01354-15) 26597982 PMC4730571

[78] Anuradha R, Munisankar S, Dolla C, Kumaran P, Nutman TB, Babu S. 2015 Parasite antigen-specific regulation of Th1, Th2, and Th17 responses in *Strongyloides stercoralis* infection. J. Immunol. **195**, 2241-2250. (10.4049/jimmunol.1500745) 26202988 PMC4546867

[79] Rajamanickam A, Munisankar S, Dolla C, Menon PA, Thiruvengadam K, Nutman TB, Babu S. 2020 Helminth infection modulates systemic pro-inflammatory cytokines and chemokines implicated in type 2 diabetes mellitus pathogenesis. PLoS Neglected Trop. Dis. **14**, e0008101. (10.1371/journal.pntd.0008101) PMC7069638 32126084

[80] Porto AF, Neva FA, Bittencourt H, Lisboa W, Thompson R, Alcantara L, Carvalho EM. 2001 HTLV-1 decreases Th2 type of immune response in patients with strongyloidiasis. Parasite Immunol. **23**, 503-507. (10.1046/j.1365-3024.2001.00407.x) 11589779

[81] Porto AF et al. 2005 Helminthic infection down-regulates type 1 immune responses in human T cell lymphotropic virus type 1 (HTLV-1) carriers and is more prevalent in HTLV-1 carriers than in patients with HTLV-1-associated myelopathy/tropical spastic paraparesis. J. Infect. Dis. **191**, 612-618. (10.1086/427560) 15655786

[82] Salles F, Bacellar A, Amorim M, Orge G, Sundberg M, Lima M, Santos S, Porto A, Carvalho E. 2013 Treatment of strongyloidiasis in HTLV-1 and *Strongyloides stercoralis* coinfected patients is associated with increased TNFα and decreased soluble IL2 receptor levels. Trans. R. Soc. Trop. Med. Hyg. **107**, 526-529. (10.1093/trstmh/trt052) 23843560 PMC3735360

[83] George PJ et al. 2015 Modulation of pro- and anti-inflammatory cytokines in active and latent tuberculosis by coexistent *Strongyloides stercoralis* infection. Tuberculosis **95**, 822-828. (10.1016/j.tube.2015.09.009) 26542223 PMC4666738

[84] Viney M, Morris R. 2022 Approaches to studying the developmental switch of *Strongyloides* – moving beyond the dauer hypothesis. Mol. Biochem. Parasitol. **249**, 111477. (10.1016/j.molbiopara.2022.111477) 35413360

[85] Gang SS, Castelletto ML, Bryant AS, Yang E, Mancuso N, Lopez JB, Pellegrini M, Hallem EA. 2017 Targeted mutagenesis in a human-parasitic nematode. PLoS Pathog. **13**, e1006675. (10.1371/journal.ppat.1006675) 29016680 PMC5650185

[86] Cadd LC, Crooks B, Marks NJ, Maule AG, Mousley A, Atkinson LE. 2022 The *Strongyloides* bioassay toolbox: a unique opportunity to accelerate functional biology for nematode parasites. Mol. Biochem. Parasitol. **252**, 111526. (10.1016/j.molbiopara.2022.111526) 36240960

[87] Mendez P, Walsh B, Hallem EA. 2022 Using newly optimized genetic tools to probe *Strongyloides* sensory behaviors. Mol. Biochem. Parasitol. **250**, 111491. (10.1016/j.molbiopara.2022.111491) 35697205 PMC9339661

[88] Tiberti N, Manfredi M, Piubelli C, Buonfrate D. 2023 Progresses and challenges in *Strongyloides* spp. proteomics. ProteomeXchange. (https://proteomecentral.proteomexchange.org/cgi/GetDataset?ID=PXD041218) 10.1098/rstb.2022.0447 PMC10676815 38008115

[89] Tiberti N, Manfredi M, Piubelli C, Buonfrate D. 2023 Progresses and challenges in *Strongyloides* spp. proteomics. Figshare. (10.6084/m9.figshare.c.6888234) PMC10676815 38008115

